# Compression Characteristics and Microscopic Mechanism of Coastal Soil Modified with Cement and Fly Ash

**DOI:** 10.3390/ma12193182

**Published:** 2019-09-28

**Authors:** Na Li, Qianying Zhu, Wei Wang, Fei Song, Dongliang An, Haoran Yan

**Affiliations:** 1 School of Civil Engineering, Shaoxing University, Shaoxing, Zhejiang 312000, China; lina@usx.edu.cn (N.L.);; 2 Department of Civil and Environmental Engineering, Technical University of Catalonia (UPC), 08034 Barcelona, Spain

**Keywords:** soft soil, fly ash, cement soil, compression characteristics, microscopic mechanism

## Abstract

It is of great significance to study the consolidation characteristics of modified coastal cement-soil. A one-dimensional consolidation test and microscopic test were carried out. In the tests, the cement content was 20%, fly ash content was 0%, 5%, 10%, 20%, and 30%, and the water content was 80%. The consolidation test results showed that: (1) Compared with coastal cement soil, the deformation of coastal cement soil modified with a 20% fly ash content was reduced from 4.31 to 2.70 mm, and the vertical compression deformation was reduced by 1.61 mm. (2) During consolidation and compression, the e–p curve (pore ratio-pressure curve) of fly ash-modified coastal cement soil was slower than that of coastal cement soil and the rate of change of pore ratio. (3) The compression coefficient of fly ash-modified coastal cement soil was reduced from 0.780 to 0.598 MPa-1 compared with that of coastal cement soil. The microscopic test results indicate that after adding the proper amount of fly ash, a skeleton was formed between the microscopic particles of the sample, which improved its resistance to compression and deformation. The results of this study indicate that it is feasible to modify coastal cement soil with an appropriate amount of fly ash to improve its compression resistance.

## 1. Introduction

Coastal soft soil refers to the sludge formed by gradual deposition under the hydrodynamic effect of weak waves and tidal waves, which are widely distributed in coastal and lake areas all over the world, especially in east China and Southeast Asia [1,2,3]. Due to its undesirable properties such as a high water content, a large pore ratio, low shear strength, and high compressibility [4,5], coastal soft soil cannot be directly used as a natural foundation. Therefore, reinforcement is generally required in practical engineering [6,7]. Coastal cement soil is a mixture of cement, water, and coastal soft soil, which is also a common soft soil reinforcement. For example, cement soil composite pile foundations and cement soil composite walls are used to enhance the strength of foundations in foundation treatment [8,9,10,11,12]. A number of studies that have aimed to improve the mechanical strength of cement soil have been carried out. Liang et al. [13] conducted experimental investigations on the mechanical behavior of cement soils with different cement contents through compression tests, and they obtained a stress–strain curve for further analysis. Through the laboratory proportioning and mechanical loading tests conducted by Chen et al. [14], the stress–strain curves of cement soil samples with five different curing agents under the age of 90 d were obtained, the effects of different curing agents and ages on the mechanical characteristics of cement soil were studied, and the relationship between compressive strength and curing age was established. An undrained triaxial compression test of cement soil was carried out by Wang et al. [15], and the test results showed that the strength and stiffness of cement soil increased with increasing confining pressure and cement content, while the pore pressure decreased.

Many studies have also been conducted on the properties of modified cement soil. Wang et al. [16] explored the effect of nano-magnesia and cement on the mechanical properties of coastal soft soil by a direct shear test, and they proposed a mathematical model for the shear stress-displacement curve. Yao et al. [11] conducted an unconfined compression test on nano-magnesia cement soil and found that the nano-magnesia had an important influence on the strength properties of the stabilized soil. Lenoir et al. [17] and Jiang et al. [18] studied the effect of fiber addition on the fatigue properties of cement soil, and they found that the addition of fibers stabilized the material properties to some extent. Asgari et al. [19] investigated the effects of cement and lime on the unconfined compressive strength of the soil. It was found that the improvement of soil mechanical properties by cement was significantly higher than that obtained from lime. In addition, the unconfined compressive pressure varied significantly with the initial water content and increasing curing time.

Compared with the prevalence of studies on unconstrained compressive strength and direct shear strength, there have been fewer studies on the consolidation characteristics of cement soil, especially modified cement soil. However, it is worth noting that due to the high compressibility of coastal soft soil, the issue of excessive deformation should not be underestimated [20,21,22,23,24]. Therefore, it is of great significance to further study the consolidation characteristics of coastal soft soil and modified coastal cement soil. The modification of mechanical strength of cement soil with fibers and nanomaterials have been studied [5,11,12,16,25,26]. However, it seems that these materials are not economical and practical because of their high cost. As a large coal-producing country, the rapid development of the power industry has brought about a sharp increase in the production of fly ash. As a common auxiliary gel material, fly ash can improve the mechanical properties of subgrade soil. It can both save project costs but protect the environment [27,28,29,30,31]. 

Recently, a micromechanism analysis put forth a solid foundation for cement matrix composite materials. Wang et al. investigated the micromechanism of nano-magnesia for improving cemented seashore soft soil and silty clay by using of the SEM and EDS (Electronic Differential System) methods [5,11,12]. Kurdi et al. integrated SEM/WDX (Wavelength dispersive X-ray spectroscopy) elemental mapping and micromorphology to determine mineralogical traits of peat soils [32]. 

The objective of this research was to take fly ash as the external admixture to modify coastal cement soil and to investigate its consolidation characteristics and microscopic mechanism under a high water content, both of which can be used as references for the reinforcement and treatment of coastal cement soil.

## 2. Test Materials 

**Figure 1:**
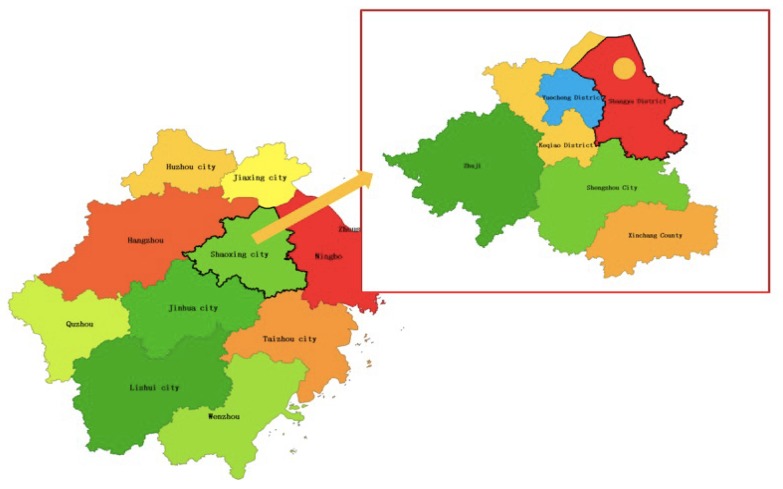
This is the location of costal soft soil.

**Figure 2:**
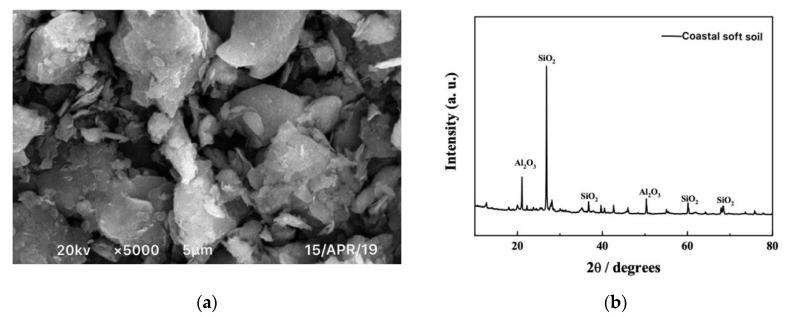
SEM and XRD analyses of coastal soft soil. (**a**) SEM of coastal soft soil; (**b**) XRD of coastal soft soil.

The coastal soft soil samples were taken from the Jiangbin area of Binhai New Town in Shangyu District, Shaoxing City, China, as shown in Figure 1. This area is located in the south wing of the Hangzhou Bay, from Qiantang River in the north and Cao’e River in the southwest. It is a typical coastal soft soil area with a high water content, which is close to rivers and lakes. Microscopic analyses using SEM (JEOL, Tokyo, Japan) and XRD (PANalytical B.V., Amsterdam, Netherlands) were carried out, and the results are shown in Figure 2. The chemical composition is summarized in Table 1. The basic physical indexes are shown in Table 2. It can be seen from Table 1 that the most important component of the coastal soft soil was SiO2, which accounted for approximately 54% of the mass of all chemical compositions; Al2O3 and MgO accounted for about 1/3 of the total mass, and there was a very small amount of NaCl.

**Figure 3:**
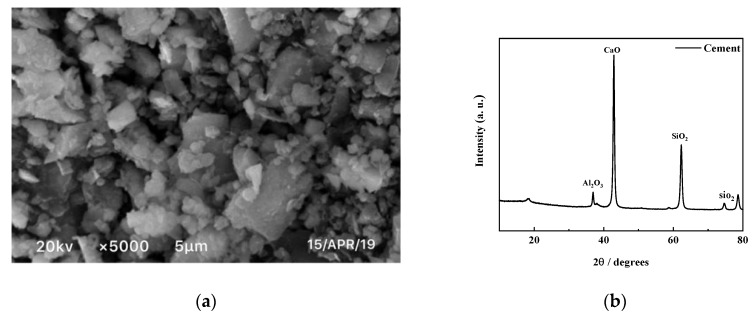
SEM and XRD results of cement. (**a**) SEM of cement; (**b**) XRD of cement.

Lanting composite Portland cement with a strength of P.O 32.5 was used in the test. The microgram of the cement surface morphology was obtained by SEM, as shown in Figure 3. It can be seen that there was little space between the surface particles, which were dense due to their tight connection. The chemical composition of cement is shown in Table 3. The main chemical composition of Portland cement was found to be CaO, which accounted for approximately 65% of the mass, followed by SiO2, which accounted for approximately 22%. The cement also contained a small amount of chemicals such as Fe2O3 and SO3.

**Figure 4:**
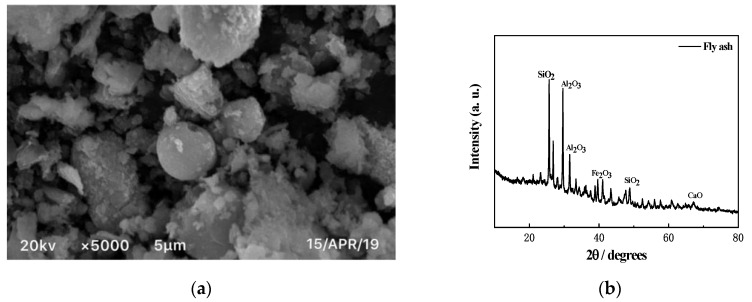
SEM and XRD of fly ash. (**a**) SEM image of fly ash; (**b**) XRD image of fly ash.

The light gray fly ash used in the test was industrial waste. SEM and XRD analyses of fly ash were obtained through micro tests and are shown in Figure 4. The chemical composition is shown in Table 4. The main chemical component of fly ash was SiO2, which accounted for about half of the total mass, and Al2O3 accounted for about one-third of the total mass.

## 3. Test Design

### 3.1. Test Methods

All coastal soft soil samples had a curing age of 7 d, 80% water content and 20% cement content. The fly ash content was varied. Five sample groups of fly ash-modified coastal cement soil were prepared with fly ash contents of 0%, 5%, 10%, 20%, and 30%. Due to the randomness of the test and the discreteness of the data, 5 samples were tested for each group of tests, that is the data were repeatedly tested five times for each group of samples. The sample content and curing age are shown in Table 5, where CF represents fly ash-modified cement soil, X represents the fly ash content, and Y represents the curing age.

### 3.2. Sample Making

The coastal soft soil samples were immersed in water for several days and sieved using 1 mm sieve. The large particles that remained in the sludge and impurities such as shells were removed, and the mixture was stirred evenly and allowed to stand for two weeks. The water content after two weeks was relatively stable. An appropriate amount of soft soil was taken to measure the actual water content.

**Figure 5:**
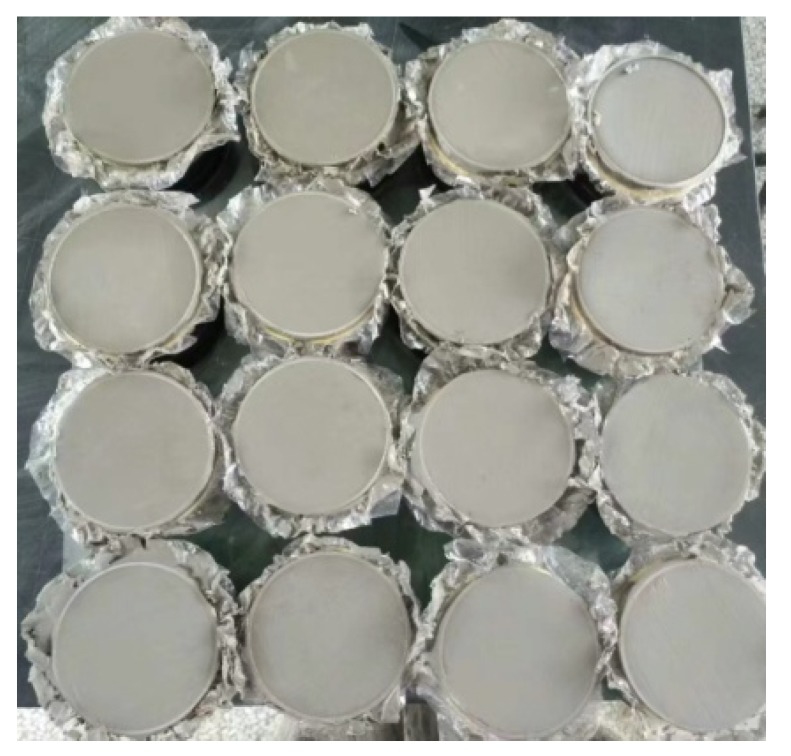
Samples for consolidation test.

The proportion was calculated according to the designed test plan, and coastal soft soil, cement, fly ash, and water were weighed, mixed, stirred evenly, and then poured into a ring cutter with a diameter of 61.8 mm and a height of 20 mm. The sample was placed in a circular ring cutter and tapped, and the surface was smoothed, as shown in Figure 5. After the sample preparation was completed, all the consolidated samples were placed in a constant temperature and humidity curing box for the required period.

### 3.3. Equipment and Load

**Figure 6:**
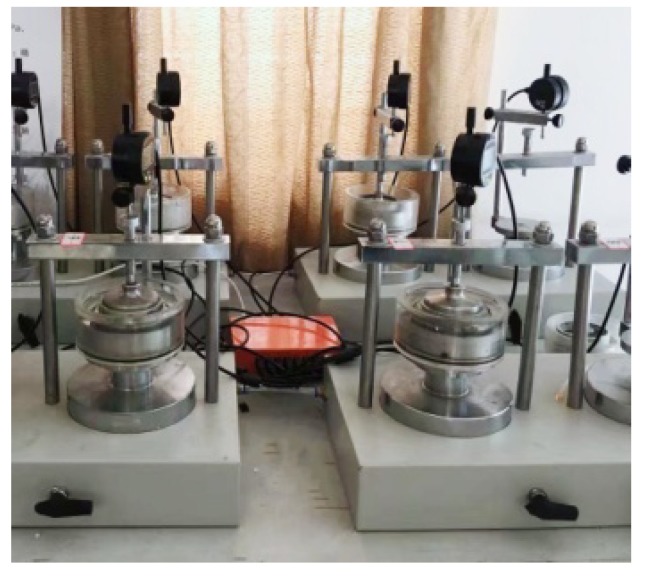
Consolidometer.

**Figure 7:**
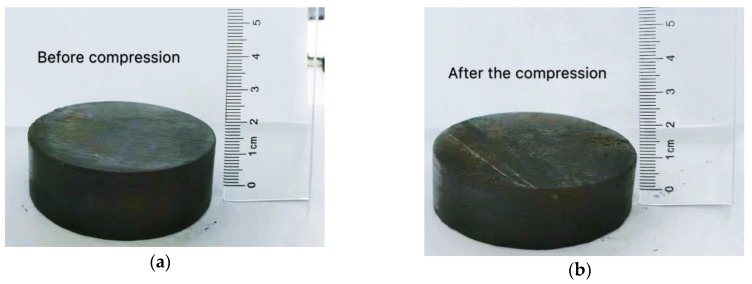
Comparison of samples before and after compression. (**a**) Sample before compression; (**b**) compressed sample.

A full-automatic pneumatic consolidometer (model No.: LH-STC-1F, TKA, Nanjing, China) was used in this test, and the pneumatic consolidation instrument used in this test is shown in Figure 6. The consolidation test was carried out in accordance with the test provisions of the geotechnical test method standard [33]. The load pressures were 12.5, 25, 50, 100, 200, 400, and 800 kPa. Considering the actual engineering conditions, the maximum load pressure was set to 800 kPa, and the pressure for each stage was maintained for 1 h. The water tank was filled and pressurized, the meter reading was adjusted, and the test was started. The comparison chart before and after compression is shown in Figure 7.

## 4. Analysis of Consolidation Compression Characteristics

### 4.1. Deformation Analysis

**Figure 8:**
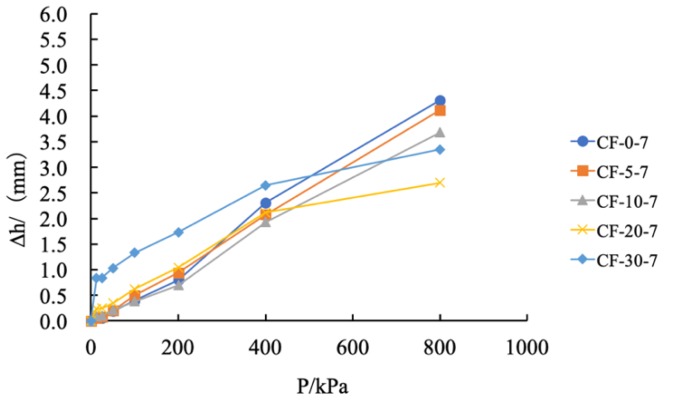
Compression deformation diagram with different fly ash content.

In order to explore the influence of the fly ash content on the vertical compression deformation of the sample, the curve between the deformation and loading pressure of coastal cement soil modified with the fly ash content of 0%, 5%, 10%, 20%, and 30% is shown in Figure 8.

**Figure 9:**
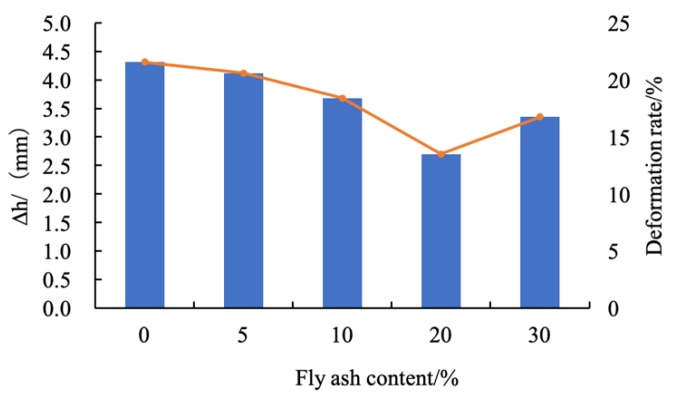
Maximum compression deformation and deformation rate with different fly ash contents.

It can be seen from Figure 8 that, as the vertical pressure increased, the vertical compression deformation also linearly increased. The maximum compressive capacity of the coastal cement soil was 4.31 mm, and the vertical compression deformation rate was 21.55%. The growth rate was fast and linear. With 5%, 10%, 20%, and 30% fly ash contents, the maximum vertical compressions were 4.12, 3.68, 2.70, and 3.35 mm, respectively, and the vertical compression deformation rates were 20.60%, 18.40%, 13.50%, and 16.75%, respectively. Corresponding to the 20% fly ash content, the growth rate of the vertical compression deformation was slow at 400 kPa; the vertical compression deformation was only 2.70 mm and the vertical deformation rate was 13.5% under a load of 800 kPa; compared with coastal cement soil, its deformation rate decreased by 8.05%. Figure 9 shows the maximum vertical compression deformation and deformation rate of fly ash-modified coastal cement soil under a load of 800 kPa.

**Figure 10:**
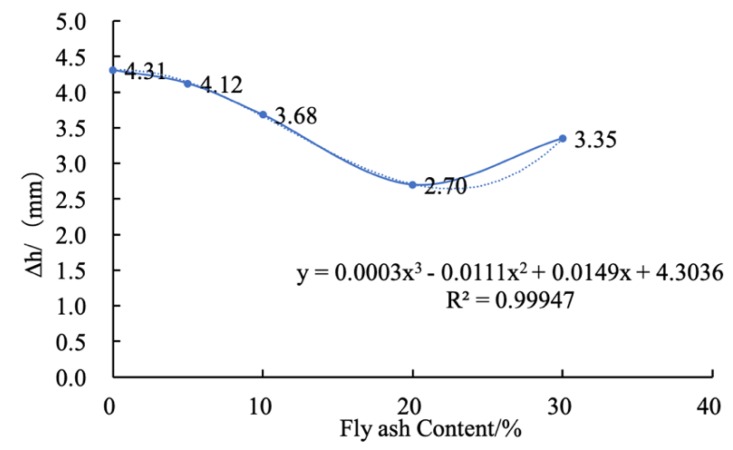
Fitting curve for fly ash content and the maximum compression deformation.

Since the fly ash content affected the maximum vertical compressive deformation of the sample, the functional relationship between the fly ash content (x%) and the maximum vertical deformation (∆h) was derived by data fitting, as shown in Figure 10 and Equation (1). In the fitting procedure, the indicator R2, i.e., the adjusted coefficient of determination [33], was introduced to evaluate the fitting accuracy. R2 is a statistical measure that shows the proportion of variation explained by the estimated regression line [34]. The closer R2 is to 1.0, the better the fitted formula explains the relationship of experimental data. When compared with the solid curves obtained by the fitted formula, the fitted formula was found to be in good agreement with the experimental data (with an adjusted coefficient of multiple determination of 0.99947).

(1) $$\Delta h=0.003X^{3}-0.011X^{2}+0.0149X+4.3036$$

It is evident form Figure 10 that was one appropriate amount of added content of fly ash into the cement soil. Adding an either bigger or smaller content than this appropriate value cannot develop good resistance to the compression of coastal cement soil. This investigation is similar to the report by Wang et al. [35].

### 4.2. Variation Analysis of Pore Ratio of Fly Ash-Modified Coastal Cement Soil

It was necessary to measure the pore ratio of the samples before the consolidation compression test of modified coastal cement soil with different fly ash contents of 0%, 5%, 10%, 20% and 30%. Referring to the measuring method of specific gravity of soil particle in soil mechanics, the particle specific gravity (ds) and sample density of coastal cement soil and fly ash-modified coastal cement soil samples after 7 d of curing were measured. According to Equation (2), the initial pore ratio of coastal cement soil and fly ash-modified coastal cement soil sample was obtained. According to Equation (3), the pore ratios of the samples under different loads were obtained. The results of the calculation are shown in Table 6.

(2) $$e_{0}=\frac{d_{s}\left(1+\omega \right)}{\rho }\rho_{\omega }-1$$

In Equation (2), d_s_ is the particle specific gravity of the sample, ω is the moisture content of the sample (%), ρ_ω_ is the density of water (g/cm^3^), and *ρ* is the density of the sample (g/cm^3^).

(3) $$e_{i}=e_{0}-\frac{1+e_{0}}{h_{0}}\Delta h$$

In Equation (3), e_0_ is the initial pore ratio of the sample, h_0_ is the initial height of the sample (mm), ∆h is the compression deformation of the sample (mm), and e_i_ is the pore ratio at a certain pressure.

**Figure 11:**
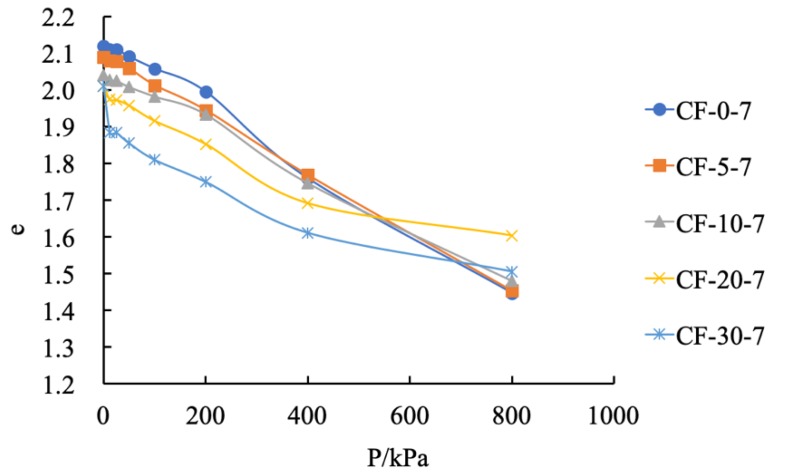
e–p curve of modified coastal cement soil.

**Figure 12:**
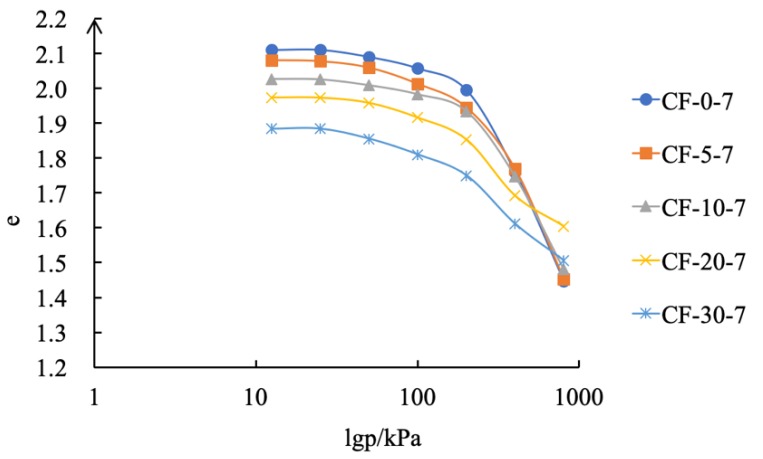
e–lgp curve of modified coastal cement-soil.

As shown in Table 6 and Figure 11 and Figure 12, the change rate of the pore ratio was also reduced after the addition of fly ash. The pore ratio was reduced by 0.67 with no fly ash content. With a fly ash contents of 5%, 10%, 20%, and 30%, the reduction in the pore ratio was only 0.64, 0.56, 0.41, and 0.50, respectively. When the fly ash content was 5% and 10%, the e–p curves of CF-5-7, CF-10-7, and coastal cement soil showed a steep trend, indicating that the pore ratio changed rapidly and the sample had high compressibility. When the fly ash content was 20% and 30%, the e–p curve slowed down, and the change rate of the pore ratio decreased, indicating that the compressibility of the sample decreased.

### 4.3. Analysis of Compression Coefficient Variation of Fly Ash-Modified Coastal Cement Soil

The compression coefficient was the ratio (MPa^−1^) of the pore ratio reduction to the increment of the effective compressive stress under the confinement condition (that is, the slope of a certain pressure section in the e–p curve), and its value varied with pressure. The more significant the pore ratio reduction, the higher the compressibility. In soil mechanics, the compressibility of soil is generally evaluated by the compression factor α_1–2_ when the pressure section p_1_ (100 kPa) is increased to p_2_ (200 kPa). Based on Equation (4), the compression coefficient of fly ash-modified coastal cement soil was calculated and analyzed.

(4) $$\alpha =\frac{e_{1}-e_{2}}{p_{2}-p_{1}}$$

In Equation (4), e_2_ is the pore ratio of the current pressure, and e_1_ is the pore ratio under the upper pressure. p_2_ (MPa)is the pressure at the current level, and p_1_ (MPa) is the upper pressure.

**Figure 13:**
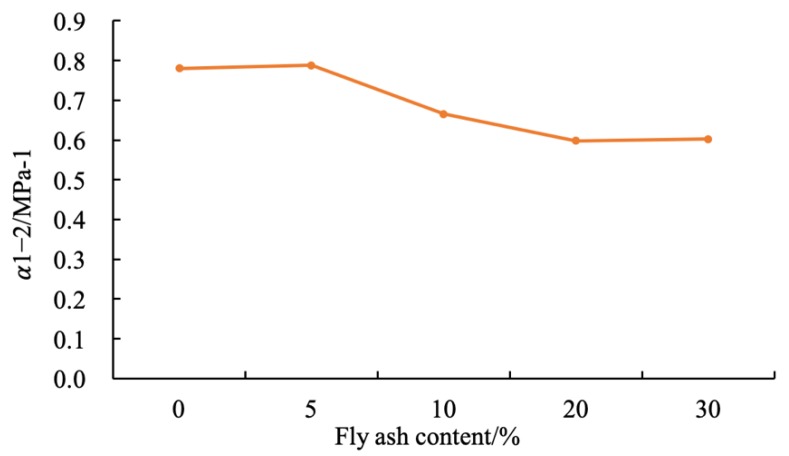
Compression coefficient of fly ash-modified coastal cement soil.

Figure 13 shows the compression coefficient of the modified coastal cement soil with different fly ash contents. Since the test samples are all with a high water content of 80%, the compression coefficients were all greater than 0.5 MPa^−1^. As can be seen from Table 7, the samples have relatively high compressibility and belong to cement-soil with high compressibility.

The compression coefficient of coastal cement soil was 0.780 MPa^−1^. After adding 5%, 10%, 20%, and 30% fly ash contents, the compression coefficients become 0.788, 0.665, 0.598, and 0.602 MPa^−1^, respectively. The 5% fly ash content had little effect on the compressive coefficient of the coastal cement soil. When the content was 10%, 20%, and 30%, the compression coefficient decreased and its compressibility reduced.

### 4.4. Relationship between Strain and Pressure Time of Modified Cement Soil

**Figure 14:**
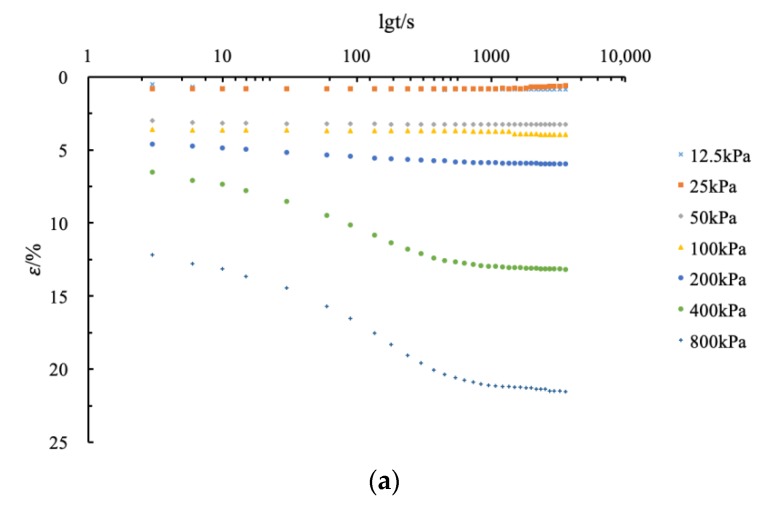

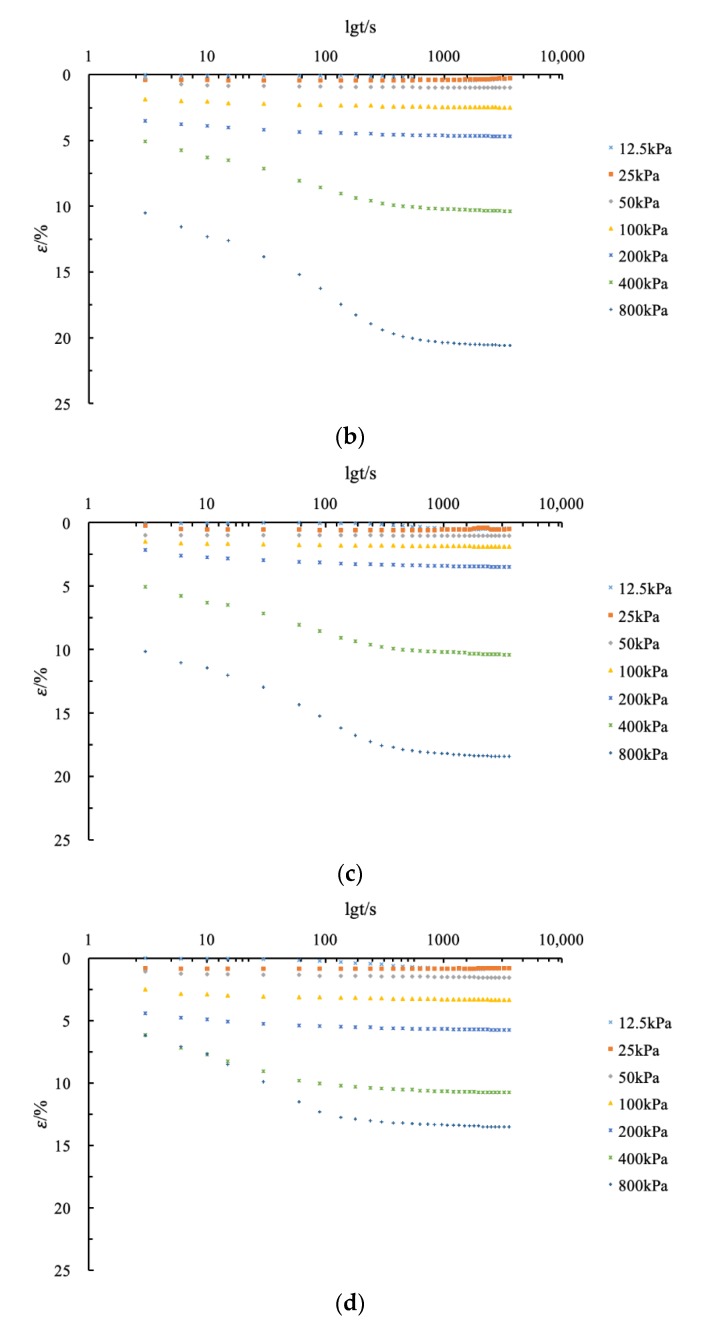

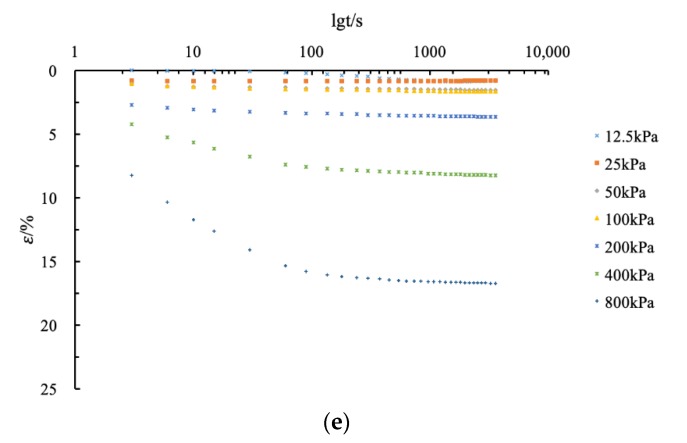
Deformation and time curve of coastal cement soil modified by different fly ash contents at 7 d. (**a**) Deformation and time curve of fly ash-modified cement soil (CF)-0-7; (**b**) deformation and time curve of CF-5-7; (**c**) deformation and time curve of CF-10-7; and (**d**) deformation and time curve of CF-20-7; (**e**) deformation and time curve of CF-30-7.

In order to investigate the relationship between the deformation and the pressurizing time under each load of fly ash-modified coastal cement soil, the deformation and time curves of CF-0-7, CF-5-7, CF-10-7, CF-20-7, and CF-30-7 are drawn as shown in Figure 14.

It can be seen from Figure 14 that the relationship between the deformation rate and time showed a nonlinear deformation characteristic. As the load increased, the nonlinear deformation characteristics of the sample increased. At the same time and under the same load, with the addition of fly ash, its deformation decreased with the increase of fly ash content. Within the first 100 s of each stage of pressure application, the deformation was not large and the sample had no significant vertical deformation. However, from 400 kPa load on, the deformation increased speedily from 100 s and tended to be stable after 1000 s. The vertical deformation rate remained basically unchanged.

## 5. Micro Analysis

### 5.1. Test Preparations

In order to further explore the anti-compression modification effect of fly ash on coastal cement soil, it was necessary to conduct a microscopic analysis, apply the obtained microscopic particle shape and skeleton structure to macroscopic mechanics, and combine macroscopic and microscopic analyses to obtain persuasive research results [32,36]. Many researchers have studied soil mass and its microstructure and tried to find the quantitative relationship between soil mass and microstructure. However, few scholars have studied the changes of micro-results of cement soil samples after adding modifiers with different contents, especially changes of their pores, element contents and compounds. Therefore, this microscopic test studied the changes of SEM, EDS and XRD after adding fly ash with different contents.

A vacuum scanning electron microscope and an energy spectrometer were used to magnify the images of the sample to 2000–5000 times by scanning electron microscopy. The SEM test was performed to obtain a micrograph of the required magnification. The elemental composition of the sample was obtained by EDS. The diffraction pattern of the sample was analyzed by XRD to elucidate its chemical composition.

**Figure 15:**
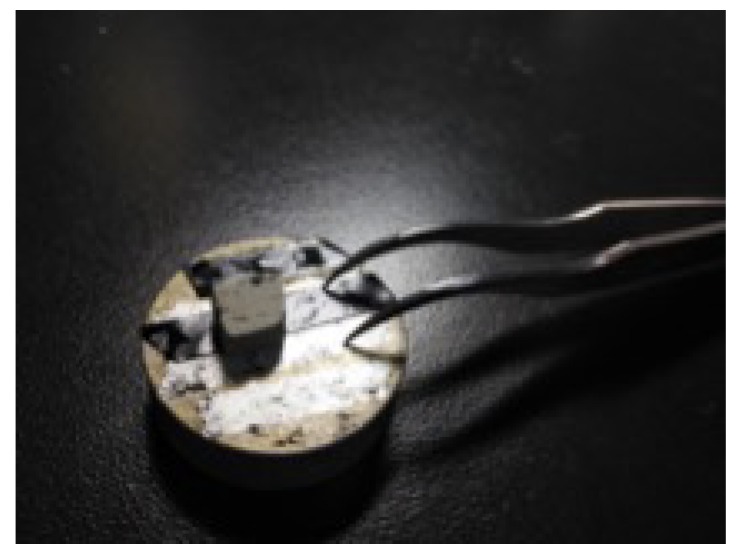
Microscopic sample preparation.

**Figure 16:**
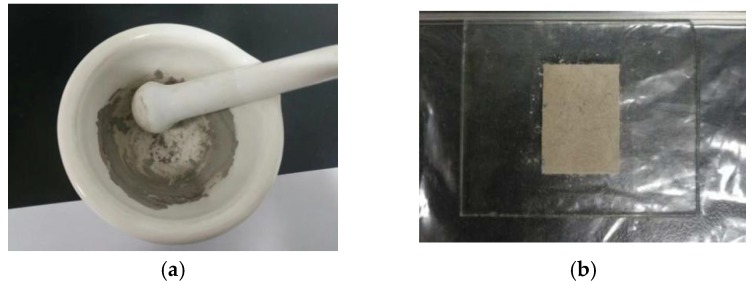
XRD sample preparation. (**a**) Ground powder; (**b**) placement in test piece.

Prior to SEM and EDS tests, cuboid micro samples of 3 mm length, 3 mm width, and 2 mm height were cut, as shown in Figure 15. The sample needed to be kept dry before the test. Natural drying was adopted in order to ensure that the sample was not damaged. For the XRD test, the sample was dried, ground into powder, and filled in a test piece, as shown in Figure 16.

### 5.2. Analysis of Test Results

In order to explore the microscopic mechanism of the resistance of fly ash-modified cement soil, SEM, EDS, and XRD micro tests of the samples of CF-0-7, CF-5-7, CF-10-7, and CF-20-7 were carried out. 

**Figure 17:**
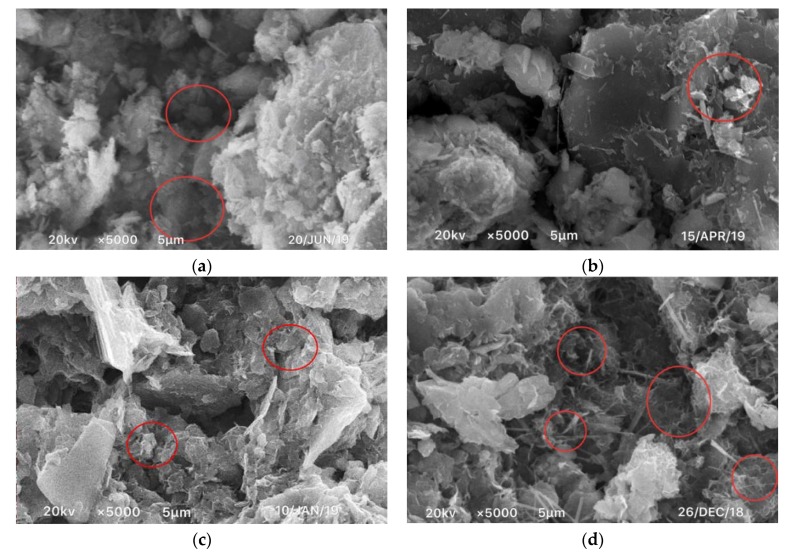
SEM of samples with different fly ash contents. (**a**) CF-0-7; (**b**) CF-5-7; (**c**) CF-10-7; and (**d**) CF-20-7.

**Figure 18:**
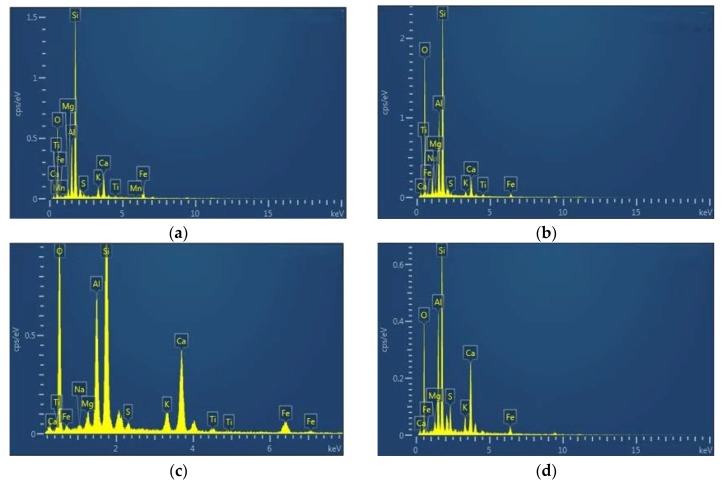
EDS images of fly ash-modified coastal cement soil. (**a**) CF-0-7; (**b**) CF-5-7; (**c**) CF-10-7; and (**d**) CF-20-7.

**Figure 19:**
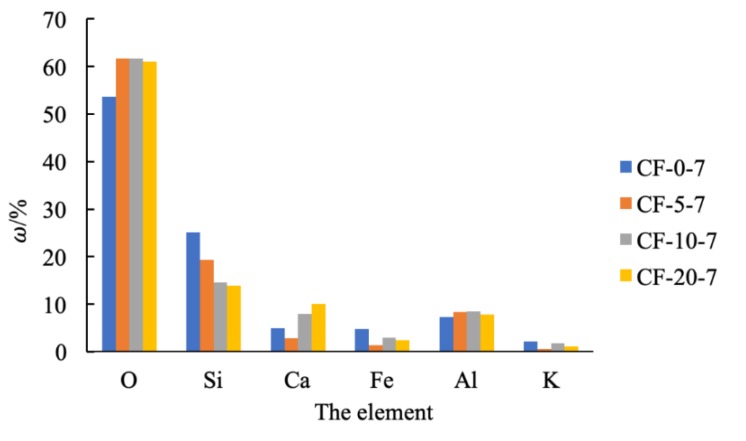
Element mass fraction of fly ash-modified coastal cement soil.

**Figure 20:**
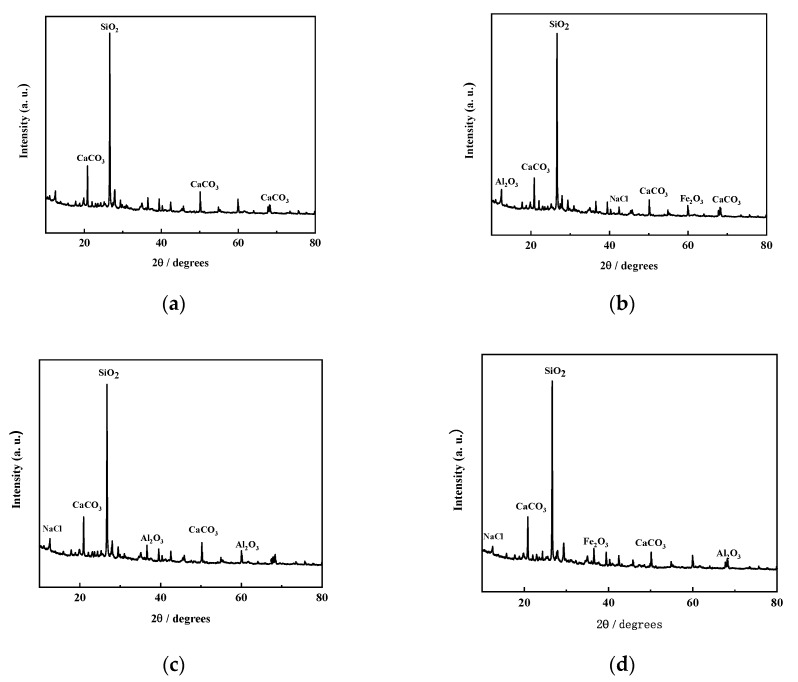
XRD of fly ash-modified coastal cement soil. (**a**) CF-0-7; (**b**) CF-5-7; (**c**) CF-10-7; and (**d**) CF-20-7.

Figure 17 shows the SEM images of CF-0-7, CF-5-7, CF-10-7, and CF-20-7. It can be seen from the images that compare coastal cement soil and fly ash-modified coastal cement soil at 7 d of age that there was flocculent hydrate attached to the particle surface of the fly ash-modified coastal cement soil. These floccules on the surface caused the soil particles to clump and form a skeleton among the pores. Therefore, a certain amount of fly ash can mechanically improve the compression resistance of coastal cement soil. Figure 17a shows the surface topography of the CF-0-7 sample. Since the sample was comprised of cement soil without fly ash, the particles and the pores between the particles were larger. With an increasing fly ash content, as shown in Figure 17b–d, the surface morphology gradually became denser and the pores were filled by the newly formed skeleton. Figure 18 shows the EDS diagram of cement soil and fly ash-modified cement soil. From of the images in Figure 18a–d, it can be seen the Ca content in the fly ash-modified coastal cement soil sample increased. Figure 19 shows the mass of different elements contained in fly ash-modified coastal cement soil with different fly ash contents and Figure 20 shows the XRD diagram of fly ash modified coastal cement soil samples with different fly ash content. As can be seen from the two pictures, with the increase in the fly ash content, elements such as O, Ca, and Al were obviously increased, which was mostly due to the incorporation of fly ash.

In summary, the hydration product of cement combined with fly ash produced a large amount of clay particles, which increased the size of the soil particles, obviously improving the soil strength. The fly ash reacted with the hydration reactant Ca(OH)_2_ of cement to form a hydrating gel [37], which created a new skeleton between the particle pores and enhanced its ability to resist deformation.

## 6. Conclusions

Based on the consolidation test data and microscopic mechanism analysis, the following conclusions can be drawn:

(1) After adding fly ash to coastal cement soil, the vertical compressive deformation and the rate of deformation decreased. Under a load of 800 kPa, the maximum vertical deformation of modified coastal cement soil with 20% fly ash content was 2.70 mm, and the deformation of coastal cement soil was 4.31 mm. The vertical compression deformation was reduced by 1.61 mm.

(2) The change rate of the pore ratio of fly ash-modified coastal cement soil also had a significant decreasing trend compared with that of coastal cement soil, which decreased from 0.67 to 0.41. The e–p curve of fly ash-modified coastal cement soil was slower than that of coastal cement soil, and the compression deformation speed slowed down.

(3) The compression coefficient of fly ash-modified coastal cement soil was also smaller than that of coastal cement soil. It could be seen that fly ash improved the deformation resistance of coastal cement soil and reduced the compressibility of the cement soil sample.

(4) Fly ash reacted with calcium ions produced by the hydration of cement to form a hydrated calcium silicate gel, which caused skeleton formation among the particle pores and improved its resistance to compression and deformation.

(5) At a short curing age, one appropriate range for added content of fly ash into cement soil existed. Only adding an appropriate fly ash content could produce good resistance to the compression and deformation behavior of coastal cement soil. In this study, the optimum fly ash content was 15%–20%. An excessive fly ash content delayed the setting time of cement, resulting in the slower hydration of cement and hence, a lack of considerable improvement of the anti-compression properties of cement soil.

It should be noted that this study described the consolidation characteristics of fly ash-modified coastal cement soil based only on the results of the consolidation compression test and microscopic test, and it explored the modification effect under a high water content and a short curing age. Further research might involve the determination of the consolidation characteristics with a different water content and a prolonged curing age. What is more, in future work, the design of experiment method should be used to highlight the influences of parameters combination.

## Figures and Tables

**Table 1:** Chemical composition of coastal soft soil.

Chemical Composition	SiO_2_	Al_2_O_3_	MgO	TiO_2_	K_2_O	Fe_2_O_3_	MnO	CaO	ZnO
ω(%)	54.05	17.34	15.78	2.07	2.36	0.85	1.08	4.43	2.05

**Table 2:** Basic physical mechanical indexes of coastal soft.

Soil	Soil Depth/m	Natural Water Content/%	Volume-Weight /g.cm^−3^	Pore Ratio	Saturability/%	Liquid Limit/%	Plastic Limit/%
Costal soft soil	1–35	67	1.63	1.74	98%	40	24

**Table 3:** Chemical composition of cement.

Chemical Composition	CaO	SiO_2_	Al_2_O_3_	Fe_2_O_3_	SO_3_
ω(%)	64.89	21.68	5.64	4.22	2.51

**Table 4:** Chemical composition of fly ash.

Chemical Composition	SiO_2_	Al_2_O_3_	Fe_2_O_3_	CaO
ω(%)	47.86	32.5	4.52	4.09

**Table 5:** Fly ash content and curing age.

Sample No.	Curing Age	Water Content	Cement Content	Fly Ash Content (%)
CF-0-7	7 d	80%	20%	0
CF-5-7				5
CF-10-7				10
CF-20-7				20
CF-30-7				30

**Table 6:** Pore ratio of samples.

Sample No.	$e_{0}$	$e_{i}$	Δe
CF-0-7	2.12	1.45	0.67
CF-5-7	2.09	1.45	0.57
CF-10-7	2.04	1.48	0.64
CF-20-7	2.01	1.60	0.41
CF-30-7	2.01	1.51	0.50

**Table 7:** Compressibility evaluation table.

$\alpha_{1-2}<0.1MPa^{-1}$	Low compressibility soil
$0.1\ll \alpha_{1-2}<0.5MPa^{-1}$	Medium compressibility soil
$\alpha_{1-2}\gg 0.5MPa^{-1}$	High compressibility soil
